# Supplementary material for: COVID-19 Vaccine Hesitancy among Italian Healthcare Workers: Latent Profiles and Their Relationships to Predictors and Outcome
Source: Vaccines (Basel). 2023 Jan 27;11(2):273. doi: 10.3390/vaccines11020273 (PMC9964484; doi:10.3390/vaccines11020273)
Supplement: Supplementary file 1 [file vaccines-11-00273-s001.zip › vaccines-2147318-supplementary.pdf]

Supplementary Material for:

COVID-19 vaccine hesitancy among Italian healthcare workers: latent profiles and their relationships to predictors and outcome

Igor Portoghese[1], Melinda Siddi[1], Luchino Chessa [1,2], Giulia Costanzo [1*], Vanessa Garcia-Larsen[3], Andrea Perra [2,4], Roberto Littera [2,5], Giada Sambugaro [1], Stefano Del Giacco [1], Marcello Campagna [1], Davide Firinu [1]

[1] Department of Medical Sciences and Public Health, University of Cagliari, 09100 Cagliari, Italy
[2] Associazione per l'Avanzamento della Ricerca per i Trapianti O.d.V., Non Profit Organisation, 09100 Cagliari, Italy
[3] Department of International Health, Bloomberg School of Public Health, The Johns Hopkins University, Baltimore, MD 21205, USA
[4] Department of Biomedical Sciences, University of Cagliari, 09100 Cagliari, Italy
[5] Medical Genetics, Department of Medical Sciences and Public Health, University of Cagliari, 09100 Cagliari, Italy
* Correspondence: Giulia Costanzo, Department of Medical Sciences and Public Health, University of Cagliari giuliacostanzo14@gmail.com +3907051096119

Supplementary Table S1: 5C Psychological Antecedents of Vaccination. Italian translation of the 5C model [14].

	Fortemente in disaccordo	*Disaccordo*	*Abbastanza in disaccordo*	*Neutrale*	*Abbastanza d'accordo*	*D'accordo*	*Fortemente d'accordo*
Sono completamente fiducioso che i vaccini siano sicuri.	1	2	3	4	5	6	7
I vaccini sono efficaci.	1	2	3	4	5	6	7
Per quanto riguarda i vaccini, sono fiducioso che le autorità pubbliche decidano nel migliore interesse della comunità.	1	2	3	4	5	6	7
La vaccinazione non è necessaria perché le malattie prevenibili con il vaccino non sono più comuni.	1	2	3	4	5	6	7
Il mio sistema immunitario è così forte che mi protegge anche dalle malattie.	1	2	3	4	5	6	7
Le malattie prevenibili con i vaccini non sono così gravi da dovermi vaccinare.	1	2	3	4	5	6	7
Lo stress quotidiano mi impedisce di vaccinarmi.	1	2	3	4	5	6	7
Per me è scomodo ricevere i vaccini.	1	2	3	4	5	6	7
Farmi visitare dal dottore mi fa sentire a disagio; questo mi impedisce di vaccinarmi.	1	2	3	4	5	6	7
Quando penso ai vaccini, valuto rischi e benefici per prendere la migliore decisione possibile.	1	2	3	4	5	6	7
Per ogni singolo vaccino, valuto attentamente se è utile per me.	1	2	3	4	5	6	7
È importante per me comprendere appieno il tema della vaccinazione, prima di essere vaccinato.	1	2	3	4	5	6	7
Se tutti sono vaccinati, non devo vaccinarmi anche io.	1	2	3	4	5	6	7

Mi vaccino perché posso proteggere anche le persone con un sistema immunitario più debole.	1	2	3	4	5	6	7
La vaccinazione è un'azione collettiva per prevenire la diffusione di malattie.	1	2	3	4	5	6	7

Supplementary Table S2: Vaccine Conspiracy Belief Scale

Italian translation of the *Vaccine Conspiracy Belief Scale* (VCBS) [27].

Per ogni frase, si prega di indicare quanto si è in disaccordo o in accordo selezionando il numero appropriato.

	Fortemente in disaccordo	*Disaccordo*	*Abbastanza in disaccordo*	*Neutrale*	*Abbastanza d'accordo*	*D'accordo*	*Fortemente d'accordo*
I dati sulla sicurezza dei vaccini sono falsificati.	1	2	3	4	5	6	7
Immunizzare i bambini è dannoso e questo fatto viene nascosto.	1	2	3	4	5	6	7
Le compagnie farmaceutiche nascondono i pericoli dei vaccini.	1	2	3	4	5	6	7
La gente viene ingannata in merito all'efficacia dei vaccini.	1	2	3	4	5	6	7
I dati sull'efficacia dei vaccini sono falsificati.	1	2	3	4	5	6	7
La gente viene ingannata in merito alla sicurezza dei vaccini.	1	2	3	4	5	6	7
Il governo sta cercando di nascondere la correlazione fra vaccini e autismo.	1	2	3	4	5	6	7
